# *Bartonella quintana* in Domestic Cat

**DOI:** 10.3201/eid1108.050101

**Published:** 2005-08

**Authors:** Vu Dang La, Lam Tran-Hung, Gérard Aboudharam, Didier Raoult, Michel Drancourt

**Affiliations:** * Université de la Méditerranée, Marseille, France

**Keywords:** Bartonella quintana, dental pulp, cat

## Abstract

We recovered *Bartonella quintana* DNA from dental pulp of a domestic cat. This study, the first to detect *B. quintana* in a nonhuman mammal, changes our understanding of the epidemiology of this infection and proposes that cats may be an emerging source of human infection.

The α-proteobacterium *Bartonella quintana* is a fastidious, gram-negative organism; humans are the only known reservoir, and the human body louse, *Pediculus humanus corporis*, is the only known vector (1). Body lice infestation is linked to poor hygiene in homeless persons and persons engaged in war, as has been reported in several circumstances since trench fever was first described during World War I. *B. quintana* causes trench fever, chronic bacteremia, and endocarditis in homeless and alcoholic patients (2) and bacillary angiomatosis in both HIV-infected and immunocompetent patients (3). Rare cases of chronic lymph node infection caused by *B. quintana* were also reported (4,5). These patients were initially diagnosed with cat-scratch disease; they lived in conditions with high hygienic standards and had no evidence of infestation by body lice; they did have close contacts with cats and flea-infested kittens, however. Similarly, the source of *B. quintana* remains unknown in a few patients with *B. quintana* bacillary angiomatosis and endocarditis. Another investigation found a 4.5% prevalence of *B. quintana* in cat fleas collected in France (6). What is missing from these puzzling cases of *B. quintana* infection, however, is documentation of *B. quintana* in a cat. In this study, by using dental pulp of domestic cats to detect *Bartonella* spp. by polymerase chain reaction (PCR) that targets fragments of the *pap31* gene, the 16S–23S internal transcribed spacer (ITS) (6,7), and 2 other genomic regions (8), we identified *B. quintana* in a cat.

## The Study

Nine domestic cats collected in Marseille were euthanized for medical indications unrelated to infectious diseases. We collected 32 cuspid teeth from these cats (Table 1), although only 1 tooth from each cat was tested for *Bartonella* DNA. Dental pulp was extracted by using an original protocol involving external decontamination by 70% ethanol and setting the entire decontaminated tooth in sterile resin (Resin Polyester Sody 33, ESCIL, Chassieu, France). After polymerization at room temperature, the apex was removed from the tooth by using a sterilized disk, and the opened canal system was inserted upside down into a sterile Eppendorf tube and centrifuged at 8,000 rpm for 10 min to recover the dental pulp. Total DNA was then extracted according to standard phenol-chloroform protocol. A negative control (sterile water) was processed in parallel exactly as described above.

**Table 1:** Results of cat tooth investigation for Bartonella spp.*

Cat	*pap31*	ITS	336	894	Sequencing results
1	+	+	NT	NT	*B. henselae* (1 mutation for *pap31*) and 100% similarity for ITS
2	+	+	+	+	*B. quintana* 100% similarity for 4 genomic regions
3	–	–	NT	NT	
4	–	–	NT	NT	
5	–	–	NT	NT	
6	–	–	NT	NT	
7	–	–	NT	NT	
8	–	–	NT	NT	
9	–	–	NT	NT	

*ITS, internal transcribed spacer; NT, not tested.

PCR amplifications were performed in a 25-μL reaction mixture containing 5 pmol of each primer (Eurogentec, Seraing, Belgium), 200 μmol/L each dNTP (Invitrogen, Cergy-Pontoise, France) in 10 mmol/L Tris-HCl (pH 8.3), 50 mmol/L KCl, 1.5 mmol/L MgCl_2_, 0.2 μg bovine serum albumin (Roche, Mannheim, Germany), 1 U Taq DNA polymerase (EuroblueTaq, Eurobio, Les Ulis, France), and 2 μL DNA. Primers PAPn1/PAPn2 targeting *pap31* were previously described (7). Primers URBarto1/URBarto2 amplified a 639-bp/722-bp ITS fragment of *B. henselae* and *B. quintana*, respectively. This fragment has 67.7% sequence similarity between *B. henselae* and *B. quintana* (6). We also amplified 2 intergenic fragments, no. 336 (597 bp) and no. 894 (383 bp), which are specific for *B. quintana* and have been incorporated into multispacer typing of *B. quintana* (8). PCR included an initial 3-min step of denaturation at 94°C followed by 41 cycles of 30 s denaturation at 94°C, 30 s primer annealing at 58°C for *pap31* primers (50°C for ITS primers), and 90 s elongation at 72°C. Amplification was completed by holding the reaction mixture at 72°C for 7 min. PCR products separated by 1.5% agarose gel electrophoresis were visualized by ethidium bromide staining, purified by using MultiScreen-PCR Filter Plate (Millipore, Saint-Quentin en Yvelines, France), and sequenced in both directions by using the d-Rhodamine Terminator Cycle Sequencing Ready Reaction kit (PerkinElmer, Coignières, France). Sequencing products were resolved in an Applied Biosystem automatic sequencer model 3100 (PerkinElmer).

**Figure 1:**
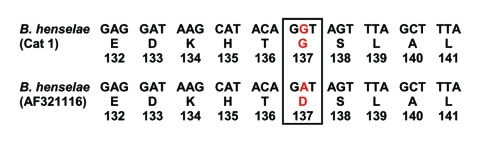
Comparison of *Bartonella henselae pap31* sequences between cat 1 and reference showing 1 mutation.

**Figure 2:**
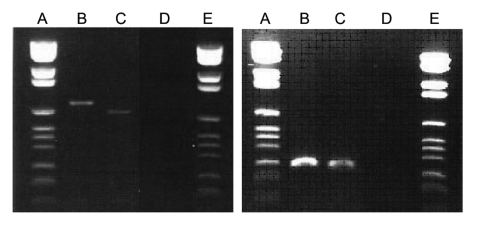
Agarose gel stained with ethidium bromide showing the amplicons intergenic spacer (left panel) and *pap31* (right panel) in cuspid teeth from 2 cats. Lanes A and E, DNA size ladder; lane B, cat 1; lane C, cat 2; lane D, negative control.

No amplification was observed for the negative controls in any PCR experiment. We obtained *pap31* amplicons with DNA extracted from the teeth of cat 1 and cat 2. A 222-bp sequence derived from the tooth of cat 2 shared complete identity with that of *B. quintana pap31* (GenBank accession no. AF308171), and a 237-bp sequence derived from the tooth of cat 1 shared 99% similarity with that of *B. henselae* ZF-1 (Houston genotype) *pap31* (GenBank accession no. AF321116). One mutation, resulting in a glycine → aspartic acid shift at codon 137, differentiated query and reference sequences (GenBank accession no. AY839861) (Figure 1). ITS amplicons obtained from the same teeth (Figure 2) shared a complete sequence identity with *B. henselae* ITS (GenBank accession no. AF312496) in cat 1 and *B. quintana* ITS (GenBank accession no. AF368395) in cat 2 (Table 1). Sequences of fragment 336 in cat 2 shared 100% similarity with *B. quintana* reference sequence (GenBank accession no. AY660705) with 3 best BLAST scores ≥1,142 (E-value 0) and 82% similarity with *B. henselae* strain Houston-1 with BLAST score of 92 (E-value 2e^–15^). Sequence of fragment 894 from the same tooth shared 100% similarity with *B. quintana* reference sequence (GenBank accession no. AY660713) with 5 best BLAST scores ≥385 (E-value ≤e^–104^).

## Conclusions

We found *B. quintana* and *B. henselae* DNA in the dental pulp of 2 domestic cats in France. To prevent contamination, we recovered pulp after the entire tooth was set in sterile resin. No amplification was obtained from controls, and no positive control was used. Amplicons were consistently obtained during separate PCR experiments targeting 4 different regions of the *Bartonella* genome. A unique mutation in the *pap31* sequence derived from a specimen definitely ruled out contamination by modern laboratory *Bartonella* DNA. We previously detected *B. henselae* DNA in dental pulp from 13th- to 16th-century domestic cats (9) and from cats buried for 1 year (10). This study is, however, the first detection of *B. henselae* ZF-1, Houston genotype outside of cat-scratch disease lymph nodes (7).

*B. quintana* identity was confirmed by amplification of 2 genomic fragments not subject to genomic transfer and by high BLAST scores with 4 different molecular targets. Until now, *B. quintana* has been detected only in humans (2,3,5) and human body lice (1). We unexpectedly recovered *B. quintana* DNA from a cat's dental pulp, which gives a prevalence of 2.5% among 39 cats tested in 3 studies, including this one (9,10). *B. henselae* was found in 23% of cats, and *B. clarridgeiae* was the most prevalent species in cat fleas. These observations agree with a 4.5% prevalence of *B. quintana* recently observed in cat fleas in France (6) (Table 2), whereas it was not detected in biting flies from California (11). We suspected as early as 1994 that cats may play a role in *B. quintana* infection (4). We described 2 patients with either *B. quintana* chronic peripheral (4) or mediastinal adenomegaly (5) who lived in good hygienic conditions and had no evidence of body lice infestation but did have close contact with cats. Ongoing PCR and sequence-based survey of lymph nodes in patients suspected of cat-scratch disease in Marseille found 11.2% *B. henselae* and 1 additional case of *B. quintana* (Table 2). A few additional patients have been reported (12). Likewise, 1 of 14 patients with *B. quintana* bacillary angiomatosis did not have risk factors, including low income, homelessness, and exposure to lice, but did have contact with cats (3,13). The same observation holds true for 3 of 38 patients with *B. quintana* endocarditis who did not have risk factors, including homelessness, alcoholism, and exposure to body lice, but did have contact with cats or cat fleas. These data led us to hypothesize that a *B. quintana* bacteremic domestic cat could be a rare source for *B. quintana* human infection. If confirmed, these data may lead to a recommendation that immunocompromised patients and patients at risk for endocarditis avoid contact with cats.

**Table 2:** Prevalence of Bartonella spp. in cats, fleas, and gland tissue from humans with suspected cat-scratch disease in Marseille

*Bartonella* sp.	Cats, n/N (%)*	Fleas, n/N (%)†	Gland tissue, n/N (%)‡
*B. henselae*	9/39 (23)	9/309 (2.9)	36/321 (11.2)
*B. clarridgeiae*	0/39 (0)	55/309 (17.8)	0/321 (0)
*B. quintana*	1/39 (2.5)	14/309 (4.5)	1/321 (0.3)


*Reference 9 and 10. †Reference 6. ‡D. Raoult, unpub. data.

Present data reinforce the idea that dental pulp is a suitable specimen on which to base PCR detection of bloodborne bacteria. In addition to our work on feline bartonellosis, we detected *B. quintana* in the dental pulp of a homeless patient with previous bacteremia (14) and in a 4,000-year-old cadaver (15). One may speculate on a common ancestor of *B. henselae* and *B. quintana* in cats, with *B. quintana* evolution toward a more specific niche. Further use of cat dental pulp to detect and genotype *B. quintana* may confirm these data and refine cat-based epidemiology and diagnosis of poorly understood clinical forms of *B. quintana* human infection.
